# Enhancing Bone Conduction Sensor Signals via Self-Supervised Acoustic Priors and Key-Value Memory

**DOI:** 10.3390/s26041137

**Published:** 2026-02-10

**Authors:** Changyan Zheng, Hao He, Xiaohu Fan, Lin Li, Yang Zhao, Ye Yan, Erwei Yin

**Affiliations:** 1 Defense Innovation Institute, Academy of Military Sciences, Beijing 100071, China; echoaimaomao@163.com (C.Z.); hehao209@126.com (H.H.); yinerwei1985@gmail.com (E.Y.); 2 High-Tech Institute, Fan Gong-ting South Street on the 12th, Weifang 261000, China; tigerlion2008@163.com (X.F.); lililin0520@sina.com (L.L.); zy940116@126.com (Y.Z.); 3 Intelligent Game and Decision Laboratory, Academy of Military Sciences, Beijing 100071, China

**Keywords:** bone conduction sensor, speech enhancement, self-supervised learning, key-value memory network

## Abstract

Bone conduction (BC) sensors naturally resist ambient noise, but the captured speech suffers from severe high-frequency attenuation due to the low-pass filtering characteristics of body tissue. To compensate for this hardware-induced information deficiency, we propose a time-domain framework leveraging highly generalized representations from Self-Supervised Learning (SSL). Specifically, we employ a large-scale pre-trained SSL model to generate embeddings that function as robust acoustic priors. Subsequently, a Key-Value Memory module is integrated to bridge the sensor domain gap, enabling the retrieval of high-fidelity priors from BC queries in the absence of reference air conduction signals. These retrieved cues are then processed by a Gated Attention Projection and dynamically fused into the primary network’s bottleneck, effectively recovering the high-frequency harmonics attenuated by the physical transmission path and rectifying the spectral distortion inherent in BC signals. Experiments on the ABCS and ESMB datasets demonstrate that our method surpasses state-of-the-art baselines in both quality and efficiency. It achieves PESQ gains of over 51% and 73% relative to raw BC inputs, respectively, with a compact architecture optimized for real-world deployment.

## 1. Introduction

Bone conduction (BC) sensors capture speech vibrations directly from body tissues and offer remarkable noise robustness in extreme scenarios such as military communications and firefighting [1]. However, the utility of these devices is often hampered by a fundamental physical bottleneck inherent to the transmission mechanism. Unlike air conduction (AC) microphones, piezoelectric or MEMS sensors must detect vibrations that have propagated through layers of skin, soft tissue, and the skull. These biological media act as severe non-linear low-pass filters and cause significant attenuation of high-frequency components above 1.5 kHz. Consequently, high-frequency formants are physically lost rather than merely corrupted. This renders conventional signal processing methods ineffective, as approaches relying on filtering or amplifying existing signals are fundamentally ill-equipped to recover information that was never effectively captured by the sensor.

In the pursuit of high-fidelity speech from such band-limited sources, two distinct research paradigms have emerged: multi-modal fusion and blind restoration. The fusion paradigm seeks to integrate synchronous AC and BC streams, capitalizing on their complementary nature through sophisticated fusion techniques [2,3,4]. In contrast, blind restoration [5] focuses on enhancing the BC signal itself. This is indispensable in scenarios where AC signals are unavailable or overwhelmed by noise, yet it is fundamentally constrained by the severe loss of the high-frequency content described above.

The core challenge in blind restoration lies in inverting the non-linear transfer function of the body conduction pathway. Deep Neural Networks (DNNs) have significantly outperformed traditional methods, such as Gaussian Mixture Models [6] and Linear Prediction Coding [7], in compensating for these channel distortions. The domain has evolved rapidly, progressing from foundational autoencoders [8] to advanced architectures leveraging attention mechanisms [9], speaker adaptation [10], and coarse-to-fine processing strategies [11]. Despite these methodological strides, a significant portion of existing work [12,13] relies on mapping spectral magnitude envelopes while inheriting the unprocessed, mechanically distorted phase from the BC sensor. To mitigate this, recent studies have shifted towards direct waveform modeling [14,15,16,17] to implicitly reconstruct phase information. However, restoring wideband fidelity from physically band-limited BC signals remains a mathematically ill-posed problem. Lacking sufficient semantic priors, purely reconstructive networks struggle to plausibly hallucinate high-frequency components that are absent due to the sensor’s hardware cut-off.

This scarcity of physical information necessitates a paradigm shift from conventional signal enhancement to cross sensor knowledge transfer. To replenish the spectral components attenuated by mechanical sensor damping, we introduce high-frequency acoustic priors derived from the AC signals. We leverage the emerging paradigm of Self-Supervised Learning (SSL) to achieve this goal. By utilizing large-scale pre-trained models such as Wav2Vec 2.0 [18], HuBERT [19], and WavLM [20], we extract embeddings from them that inherently encapsulate universal acoustic and linguistic priors. We hypothesize that these information-rich vectors function as high-fidelity restoration prompts, providing the necessary structural guidance to plausibly recover high-frequency details that the BC sensor failed to capture. However, the deployment of these SSL models presents a fundamental dilemma because the reference AC signals required to generate prompts are unavailable during the inference phase. To bridge this sensor domain gap, we design a Key-Value Memory Network that functions as an associative retrieval mechanism. This allows the system to store high-fidelity AC priors during training and recall them using BC queries in deployment.

To address the severe signal attenuation caused by the physical limitations of BC sensors, we propose a novel time-domain framework for BC speech enhancement. The main contributions of this paper are summarized as follows:

- Cross-Sensor Knowledge Transfer Framework: To the best of our knowledge, this is the first work to integrate large-scale SSL models into BC speech enhancement. By transferring high-fidelity acoustic priors from SSL embeddings, our method mitigates the hardware-imposed bandwidth limitation of BC sensors, significantly enhancing the fidelity and intelligibility of the sensor output and restoring fine-grained high-frequency content. Audio samples are publicly available at https://echoaimaomao.github.io/LeverageWav2Vec/ (accessed on 1 February 2026).
- Reference-free Retrieval via Key-Value Memory: We introduce a Key-Value Memory module to bridge the gap between BC and AC sensor signal domains. By mapping BC queries to robust acoustic priors, this mechanism retrieves high-fidelity restoration prompts during inference using solely BC input. This architecture also eliminates the need to execute the large SSL models during deployment, effectively reducing computational overhead for resource-constrained devices.
- Flexible Plug-and-Play Adaptor Design: We employ a lightweight Gated Attention Projection and cross-attention mechanism to dynamically align and fuse the retrieved priors with the backbone features. This design decouples the restoration network from the knowledge source, establishing a framework where both the backbone and the pre-trained model can be flexibly replaced or upgraded. It is worth noting that the computational overhead of the large-scale pre-trained model is strictly confined to the training phase, ensuring that the inference model remains lightweight and efficient for deployment on resource-constrained devices.

## 2. Background and Related Work

### 2.1. Signal Model

Based on the classical source-filter theory and physiological studies on bone conduction [21], both AC and BC signals originate from the same physiological excitation e(t) generated by vocal cord vibration. However, they traverse distinct transmission pathways before being captured. Following the signal formulation in [22], we model the captured AC signal $x_{ac}\left(t\right)$ and BC signal $x_{bc}\left(t\right)$ as the convolution of the source excitation and their respective path responses:

(1) $$x_{ac}\left(t\right)=e\left(t\right)*h_{ac}\left(t\right)+n_{ac}\left(t\right),$$

(2) $$x_{bc}\left(t\right)=e\left(t\right)*h_{bc}\left(t\right)+n_{bc}\left(t\right),$$

where ∗ denotes convolution. Here, $h_{ac}\left(t\right)$ represents the acoustic path response. In contrast, $h_{bc}\left(t\right)$ models the osteo-conductive path through the skull and soft tissues. As analyzed in [21,23], the impedance of skin and tissue acts as a complex non-linear filter, resulting in the significant attenuation of high-frequency spectral components observed in BC speech recordings [24]. $n_{ac}\left(t\right)$ and $n_{bc}\left(t\right)$ denote ambient and sensor noise, respectively.

Given that BC sensors generally exhibit strong insensitivity to ambient air-conducted noise, the primary degradation in BC speech manifests as bandwidth limitation rather than noise contamination. Therefore, focusing on the spectral characteristics and neglecting the noise terms for the mapping formulation, we model the relationship from the high-fidelity AC domain to the band-limited BC domain as a non-linear channel transformation T:

(3) $$x_{bc}\left(t\right)\approx T\left(x_{ac}\left(t\right)\right)$$

Consequently, the objective of blind BC enhancement is to approximate the inverse transformation using a deep parametric function F:

(4) $${\hat{x}}_{ac}\left(t\right)=F\left(x_{bc}\left(t\right)\right)\approx T^{-1}\left(x_{bc}\left(t\right)\right)$$

Since T incurs irreversible physical information loss, finding the analytical inverse $T^{-1}$ is mathematically ill-posed. This inherent ambiguity necessitates the integration of external acoustic priors to regularize the inversion process and guide the reconstruction of missing spectral details.

### 2.2. Bone Conduction Speech Enhancement

Early research on compensating for the spectral limitations of BC sensors relied on statistical methods such as Gaussian Mixture Models [25] and Linear Prediction Coding [26]. While these approaches established a theoretical foundation, they often failed to recover fine-grained spectral details from the severely band-limited inputs typical of piezoelectric or MEMS sensors. Deep learning approaches subsequently emerged to address these hardware constraints. Frequency-domain models achieved spectral reconstruction but often suffered from phase mismatch due to the reliance on unprocessed BC phase information. To mitigate this, recent time-domain architectures like DPT-EGNet [15], EBEN [16] and U-Net-like [17] enable joint optimization of magnitude and phase. Our work builds upon this time-domain paradigm. However, relying solely on the band-limited sensor input makes wideband recovery mathematically ill-posed. This necessitates the integration of external acoustic knowledge to compensate for the hardware-induced information scarcity.

### 2.3. Self-Supervised Learning in Speech Processing

Self-Supervised Learning (SSL) has revolutionized speech processing by shifting the paradigm from supervised training on limited labeled data to learning from vast amounts of unlabeled audio [27]. The core philosophy typically involves a mask-and-predict mechanism, where the model is tasked with reconstructing or identifying hidden parts of the input based on the remaining context. This pretext task forces the network to capture latent acoustic structures and long-range temporal dependencies.

Several representative frameworks exemplify this paradigm. Wav2Vec 2.0 [18] relies on contrastive learning, masking latent representations of raw audio and training the model to distinguish the true quantized representation of the masked time step from a set of distractors. HuBERT [19] shifts to a prediction-based approach analogous to BERT in NLP. It utilizes offline clustering to generate discrete pseudo-labels and optimizes the model to predict these cluster assignments from masked inputs, effectively mapping continuous signals into discrete phonetic-like units. Building upon the HuBERT architecture, WavLM [20] introduces a masked speech denoising modeling task. It mixes noise or overlapping speech into the input while requiring the model to predict the pseudo-labels of the original clean speech. This denoising objective enables WavLM to learn representations that are not only phonetically rich but also inherently robust to complex acoustic environments and background noise.

These SSL objectives enable models to encode rich linguistic and paralinguistic information, including phonemes, prosody, and speaker identity, without human annotation. Consequently, the resulting embeddings serve as highly generalized features that have proven effective in various downstream tasks, such as Automatic Speech Recognition [28], Speech Emotion Recognition [29], and Voice Conversion [30]. In the context of signals characterized by severe information scarcity, these SSL-derived priors are particularly advantageous. They provide a robust structural foundation of linguistic and acoustic consistency, effectively compensating for the physical signal loss that limits the efficacy of traditional signal processing methods. However, to the best of our knowledge, the utilization of these powerful pre-trained representations, particularly as cross-modal priors, remains unexplored in the field of BC speech enhancement.

### 2.4. Key-Value Memory Networks for Cross Modal Retrieval

To address the absence of reference AC signals during inference, we draw inspiration from Key-Value Memory Networks, which have proven highly effective in cross-modal retrieval and generation tasks. Unlike direct mapping approaches, this architecture introduces an explicit storage mechanism to associate heterogeneous modalities, making it particularly suitable for scenarios where one modality is missing.

This paradigm has been extensively validated in the field of Visual Speech Recognition (VSR). For instance, memory networks have been successfully employed to reconstruct missing acoustic features solely from visual lip movements [31,32,33]. In these frameworks, the memory acts as a bridge, storing representative audio prototypes that can be queried by visual features to synthesize coherent speech. Drawing parallels to this visual-to-audio mapping, we adapt the Key-Value Memory Network to the domain of BC speech enhancement. By treating BC features as keys and high-fidelity acoustic priors extracted from SSL models as values, our proposed method enables the associative retrieval of clean speech representations even when the AC signal is unavailable. This mechanism effectively acts as a domain bridge, translating the band-limited BC feature space into the high-fidelity AC feature space via a learned codebook, thereby solving the missing reference problem inherent to blind restoration tasks.

## 3. Methodology

To effectively recover lost spectral content, we design a time-domain framework that integrates generalized acoustic knowledge from self-supervised learning to compensate for the information scarcity in BC signals. Considering the unique challenges of BC enhancement, specifically the severe non-linear high-frequency attenuation and the lack of clean references during inference, we integrate three synergistic strategies. First, we adopt Wave-U-Net [34] as the backbone for its phase-aware time-domain modeling capabilities, avoiding the phase estimation errors common in frequency-domain approaches. Second, to address the information bottleneck where the sensor physically loses high-frequency harmonics, we introduce SSL representations as semantic anchors. Unlike traditional supervised features, SSL priors encapsulate rich universal acoustic structures, enabling the model to infer plausible high-frequency content even from severely band-limited inputs. Third, to bridge the domain gap between the distorted BC signal and clean AC priors, we employ the Key-Value Memory mechanism described above. This allows for the associative retrieval of high-fidelity restoration cues during inference without requiring paired AC inputs.

**Figure 1:**
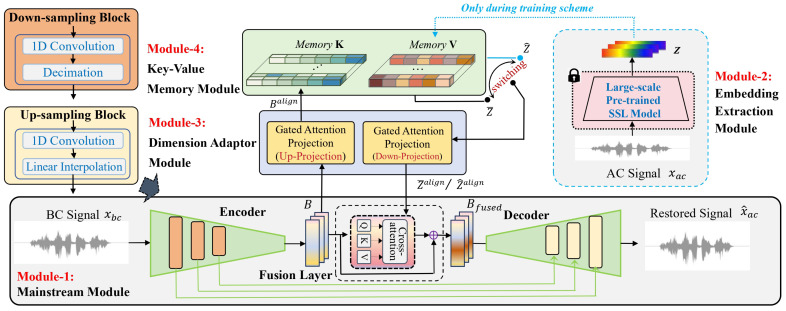
The framework of the proposed model. The Mainstream Module is responsible for generating restored speech from BC signals. In the training stage, embeddings are derived from the Embedding Extraction Module and are dimensionally aligned by the Dimension Adaptor Module. The Key-Value Memory Module mimics the high-quality embeddings during training and recalls them to guide speech decoding during inference.

As illustrated in Figure 1, the system comprises four synergistic components:

- Mainstream Module that serves as the backbone for feature encoding and waveform reconstruction;
- Embedding Extraction Module that utilizes large-scale pre-trained SSL model to extract embeddings encapsulating high-fidelity acoustic priors;
- Dimension Adaptor Module specifically designed to align the dimensional discrepancy between bottleneck features and external embeddings via Up- and Down-Projection operations;
- Key-Value Memory Module that bridges the modality gap, enabling the associative retrieval of these idealized priors using BC features as queries.

It is worth noting that the Embedding Extraction Module is employed exclusively during the training phase to provide supplementary restoration cues and is detached during inference.

### 3.1. The Mainstream Module

Serving as the backbone for waveform reconstruction, the Mainstream Module is built upon the Wave-U-Net architecture. Selected for its robust time-domain modeling capabilities, this network effectively captures multi-scale temporal context, a property critical for BC speech restoration, as validated in prior work [17].

As illustrated in Figure 1, the module adopts a symmetric encoder–decoder structure with skip connections. The encoder comprises a series of Down-sampling blocks that progressively reduce the temporal resolution of the input while increasing channel capacity. Each block consists of a 1D convolution layer followed by a decimation operation. This hierarchical feature extraction culminates in a low-dimensional bottleneck representation, denoted as $B\in R^{T\times C}$, where *T* represents the compressed time steps and *C* denotes the channel dimensionality. Conversely, the decoder employs Up-sampling blocks to restore the temporal resolution, fusing features from the encoder via skip connections to recover fine-grained details.

To tailor the Wave-U-Net to our specific task, we introduced several modifications. The original multi-head output designed for source separation was consolidated into a single regression head for mono speech restoration. Furthermore, the network depth and the base number of convolutional channels were optimized to balance restoration performance with computational complexity.

Table 1 presents the detailed architecture of the Mainstream Module. Notably, the network configuration is provided for both 1 s and 2 s input durations to accommodate our two experimental datasets. All audio segments are processed at a sampling rate of 16 kHz, with the base channel count set to n=25. In the *Operation* column, Conv1D(*x*) denotes a 1D convolution with *x* output filters. The *Decimation* operation halves the temporal resolution by discarding alternate time steps, whereas the *Upsample* operation doubles the resolution via linear interpolation. Further implementation details align with the original Wave-U-Net strategy.

**Figure 2:**
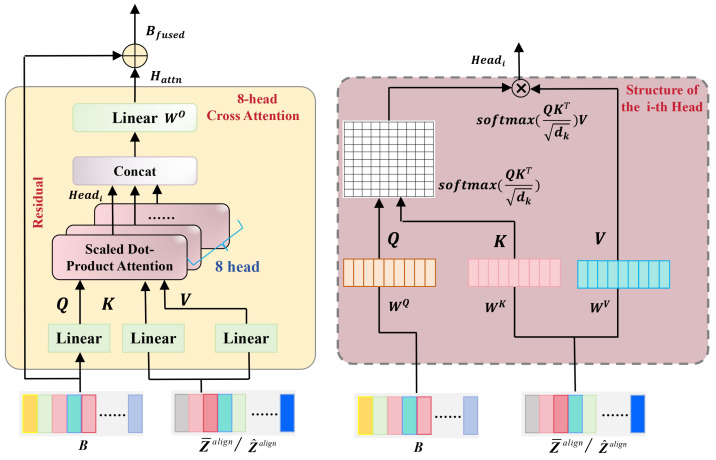
Detailed architecture of the Cross-Attention-based Fusion Layer. The bottleneck feature *B* from the Mainstream encoder serves as the Query Q, while the aligned embeddings $Z^{align}$ retrieved from the memory module function as both Key K and Value V. A residual connection adds the attention-weighted external priors back to the original bottleneck features, enabling the dynamic integration of semantic guidance while preserving the original information.

To effectively incorporate external acoustic priors into the reconstruction process, we augment the bottleneck with a cross-modal fusion mechanism. As illustrated in Figure 1, a Cross-Attention-based fusion layer is positioned between the encoder and decoder. This layer dynamically integrates the bottleneck features *B* with external restoration prompts from the Key-Value Memory Module while preserving the original signal flow via a residual connection. The detailed internal structure is shown in Figure 2.

Let $Z^{align}$ denote the aligned embeddings retrieved from the memory module (corresponding to ${\bar{Z}}^{align}$ or ${\hat{Z}}^{align}$ in Figure 1). In the cross-attention calculation, the BC bottleneck feature *B* functions as the query, requesting relevant information from the external embedding $Z^{align}$, which serves as both the key and value. Mathematically, inputs are projected into subspace representations as follows:

(5) $$Q=BW^{Q},\;K=Z^{align}W^{K},\;V=Z^{align}W^{V},$$

where $W^{Q}$, $W^{K}$, and $W^{V}$ are learnable projection matrices. To capture diverse semantic aspects, we employ Multi-Head Attention (MHA) with h=8 parallel heads. For the *i*-th head, the scaled dot-product attention is computed as:

(6) $${\mathrm{Head}}_{i}=\mathrm{Attention}\left(Q_{i},K_{i},V_{i}\right)=\mathrm{softmax}\left(\frac{Q_{i}K_{i}^{T}}{\sqrt{d_{k}}}\right)V_{i},$$

where $d_{k}$ acts as the scaling factor. Subsequently, the outputs from all *h* heads are concatenated and fused via a linear projection $W^{O}$ to produce the attention output $H_{\mathrm{attn}}$:

(7) $$H_{\mathrm{attn}}=\mathrm{Concat}\left({\mathrm{Head}}_{1},\ldots ,{\mathrm{Head}}_{h}\right)W^{O},$$

The final fused representation $B_{\mathrm{fused}}$ is obtained by adding the residual term to the original bottleneck:

(8) $$B_{\mathrm{fused}}=B+H_{\mathrm{attn}}.$$

By leveraging this residual mechanism, $B_{\mathrm{fused}}$ retains the structural integrity of the BC speech while selectively assimilating high-fidelity acoustic details from external priors, thereby guiding fine-grained reconstruction in the decoder.

### 3.2. Embedding Extraction Module

The objective of the Embedding Extraction Module is to capture high-fidelity acoustic priors from reference AC signals. As depicted in Module-2 of Figure 1, this module functions as a feature extractor that transforms raw waveforms into generalized semantic representations to provide a ground-truth blueprint for training the Memory Module.

Formally, let $x_{ac}\in R^{L}$ denote the input reference AC signal waveform of length *L*. We employ a pre-trained and frozen self-supervised model such as Wav2Vec 2.0 or HuBERT as the feature extractor, denoted by the function $F_{SSL}$. The extraction process is formulated as:

(9) $$Z=F_{SSL}\left(x_{ac}\right),$$

where $Z\in R^{T^{\prime }\times D}$ represents the extracted embedding sequence with downsampled time steps $T^{\prime }$ and hidden dimension *D*. These embeddings *Z* serve as the target values for constructing the memory space.

Structurally, these frameworks typically comprise a multi-layer convolutional feature encoder followed by a Transformer context network. It is well established that the encoded information varies significantly across these layers. Lower layers tend to retain fine-grained acoustic details, whereas upper layers encapsulate richer semantic and linguistic content. Consequently, the optimal representation *Z* depends on the specific source layer within the model. We systematically investigate the optimal selection of the pre-trained model in our experiments.

### 3.3. Dimension Adaptor Module

The distinct architectures of the Mainstream Module and the external SSL model create a significant dimensional discrepancy. Specifically, the bottleneck feature *B* from the Wave-U-Net typically possesses a compact channel dimensionality to enforce information compression. In contrast, SSL models output semantic embeddings with significantly higher dimensions. To resolve this mismatch and facilitate the flexible integration of external priors, we design the Dimension Adaptor Module.

**Figure 3:**
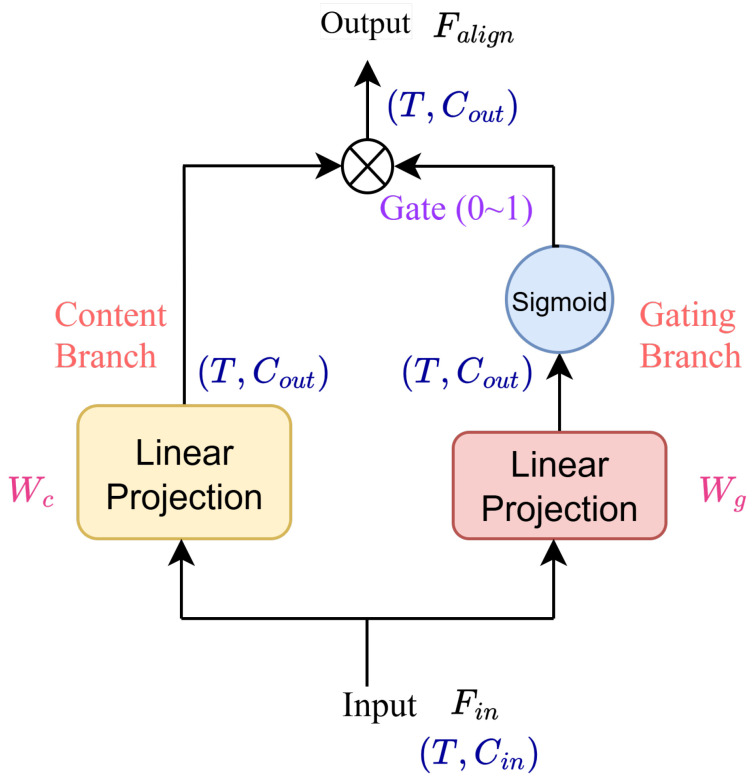
Structure of the Gated Attention Projection. This module aligns the dimensional discrepancy between the low-dimensional bottleneck features and high-dimensional SSL embeddings. It employs a dual-path design: a Content Branch for feature projection and a Gating Branch to generate a soft mask. This mechanism acts as a dynamic filter, selectively enhancing salient acoustic features while suppressing noise during the dimension mapping process.

As illustrated in Figure 1, this module is composed of two symmetric sub-blocks: (1) an Up-Projection, which maps the low-dimensional bottleneck features into the high-dimensional embedding space to query the memory module; and (2) a Down-Projection, which compresses the embedding features back to the bottleneck dimension for waveform reconstruction. To enhance the feature transformation capability beyond simple linear mapping, we employ a Gated Linear Unit mechanism, which we term the *Gated Attention Projection*. The internal structure is detailed in Figure 3.

Let $F_{in}\in R^{T\times C_{in}}$ denote the input feature, where $C_{in}$ represents the input channel dimension. The adaptor splits the information flow into two parallel paths: a Content Branch and a Gating Branch. Mathematically, the operation is formulated as:

(10) $$F_{align}=\underset{\mathrm{Content}}{\underset{︸}{\left(F_{in}W_{c}\right)}}⊙\underset{\mathrm{Gate}}{\underset{︸}{\sigma (F_{in}W_{g})}},$$

where $F_{align}\in R^{T\times C_{out}}$ is the output feature. $W_{c}\in R^{C_{in}\times C_{out}}$ and $W_{g}\in R^{C_{in}\times C_{out}}$ denote the learnable weight matrices for the content projection and gate projection, respectively. The symbol ⊙ represents the element-wise Hadamard product, and σ(·) denotes the Sigmoid activation function. As depicted in Figure 1, this module transforms *B* to $B^{align}$, $\hat{Z}$ to ${\hat{Z}}^{align}$, and $\bar{Z}$ to ${\bar{Z}}^{align}$.

By decoupling the feature projection from the information regulation, this design allows the Content Branch to handle the dimensional scaling, while the Gating Branch simultaneously produces a soft mask (0,1) to modulate the flow. This acts as a dynamic filter that effectively suppresses noise and enhances salient acoustic features, offering a more robust attention-like alignment mechanism than standard fully connected layers.

### 3.4. Key-Value Memory Module

To address the critical challenge where the reference AC signal is unavailable during inference, we introduce the Key-Value Memory Module. Its primary role is to serve as a cross-modal dictionary that bridges the domain gap between distorted BC speech and clean AC representations.

**Figure 4:**
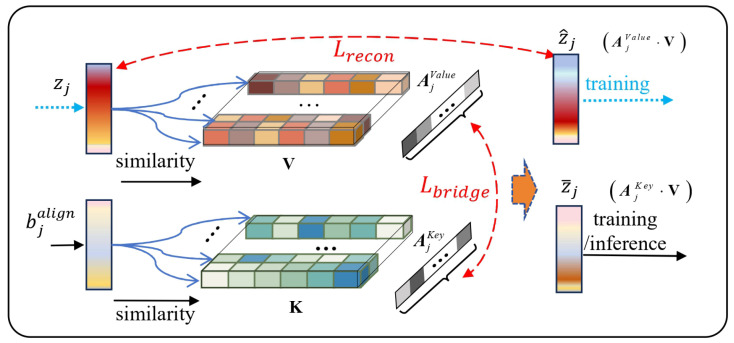
Illustration of the Key-Value Memory Module. This module consists of a key memory and a value memory. During training, the AC embedding $z_{j}$ is utilized to update value memory V via $L_{recon}$, while $L_{bridge}$ aligns the addressing vectors of both memories. This mechanism allows the recalling priors ${\bar{z}}_{j}$ using only the BC input $b_{j}^{align}$ during inference. Dashed arrows indicate the loss calculation paths used specifically during the training phase.

As illustrated in Figure 4, the module comprises a key memory K and a value memory V, both implemented as trainable data matrices. V stores the high-fidelity acoustic embeddings extracted by the SSL model, while K serves as a shared addressing basis. By aligning the addressing distributions on this shared basis, a reliable connection is established between the heterogeneous modalities. Consequently, this design enables the system to utilize BC features as queries to retrieve the optimal acoustic priors from V in the absence of AC speech.

#### 3.4.1. Storing and Addressing Representative Features

To store the representative features, the similarity between the features inside V and the embedding *Z* is evaluated. For each incoming embedding $z_{j}$ at timestep *j*, we compute its cosine similarity with every slot $v_{i}$ in V. Subsequently, a Softmax normalization is applied to these metrics to yield a probability distribution, effectively functioning as the addressing vector for memory retrieval. The equations are as follows:

(11) $$s_{i,j}^{value}=\frac{v_{i}\cdot z_{j}}{\left|\right|v_{i}{\left|\right|}_{2}\cdot \left|\right|z_{j}{\left|\right|}_{2}},$$

(12) $$a_{i,j}^{value}=\frac{exp(\tau \cdot s_{i,j}^{value})}{\sum_{k=1}^{N}exp\left(\tau \cdot s_{k,j}^{value}\right)},$$

where *N* is the number of slots in V, and τ is the temperature constant used to control the sparsity of Softmax distribution.

By computing this probability distribution over all *N* slots, we obtain the addressing vector for the value memory, $A_{j}^{Value}=\left\{a_{1,j}^{value},a_{2,j}^{value},\cdots ,a_{N,j}^{value}\right\}$. An identical procedure, using the aligned BC bottleneck feature $b_{j}^{align}$ as input, is used to obtain the addressing vector for the key memory, $A_{j}^{Key}$.

#### 3.4.2. Bridging the Two Memories

V is trained to memorize *Z*. The reconstructed embedding at timestep *j* is obtained as follows:

(13) $${\hat{z}}_{j}=A_{j}^{Value}\cdot V,$$

Then, the reconstruction loss function is used to guide V to save the proper representation,

(14) $$L_{recon}=E_{j}\left[|\right|z_{j}-{\hat{z}}_{j}{\left|\right|}_{2}^{2}],$$

where $z_{j}$ is the target instant embedding. Since $A_{j}^{Value}$ cannot be provided in the inference stage, $A_{j}^{Key}$ is guided to match it with the following bridging loss:

(15) $$L_{bridge}=E_{j}[D_{KL}(A_{j}^{Value}\left|\right|A_{j}^{Key})],$$

where $D_{KL}\left(\cdot \right)$ represents Kullback–Leibler divergence.

#### 3.4.3. Recalling the Target Embeddings

Through the associative bridge, $A_{j}^{Key}$ provides location information for the corresponding saved embeddings in V. Therefore, the recalled embedding ${\bar{z}}_{j}$ can be obtained as follows:

(16) $${\bar{z}}_{j}=A_{j}^{Key}\cdot V,$$

### 3.5. The Objective Function

The proposed framework is optimized in an end-to-end manner. Since the ultimate goal is to generate a waveform that perceptually and spectrally approximates natural AC speech, we define a task-specific loss to measure the discrepancy between the restored output and the ground-truth target $x_{ac}$:

(17) $$L_{task}=g\left(h\left(B,{\hat{Z}}^{align}\right);x_{ac}\right)+g\left(h\left(B,{\bar{Z}}^{align}\right);x_{ac}\right),$$

where h(·) denotes the composite function of the fusion layer and the decoder, and g(·) represents the multi-scale spectral loss function adopted from [17].

Crucially, this loss comprises two terms: the first term utilizes the aligned ground-truth embedding ${\hat{Z}}^{align}$ to ensure that meaningful and accurate representations are stored in V; the second term utilizes the recalled embedding ${\bar{Z}}^{align}$ to simulate the actual inference scenario. This dual-term design ensures that the model can effectively fuse the bottleneck features of BC speech with the recalled priors to restore the missing high-frequency components. Finally, the total objective function is formulated as a weighted sum of the component losses:

(18) $$L_{total}=\lambda_{1}L_{task}+\lambda_{2}L_{recon}+\lambda_{3}L_{bridge}.$$

Balancing these terms is critical to prevent any single loss from dominating the gradient optimization. We empirically adjust the hyperparameters to ensure comparable magnitudes, setting $\lambda_{1}=1$ and $\lambda_{2}=\lambda_{3}=250$ in our experiments.

## 4. Experimental Setup

### 4.1. Datasets and Metrics

#### 4.1.1. Datasets

We conduct experiments on two public 16 kHz datasets: the Air- and Bone-conduction Synchronized (ABCS) corpus (Available: https://github.com/wangmou21/abcs accessed on 1 February 2026) and the Elevoc Simultaneously recorded Microphone/Bone-sensor (ESMB) corpus (Available: https://github.com/elevoctech/ESMB-corpus accessed on 1 February 2026). In both datasets, high-fidelity AC speech is recorded simultaneously with the BC signal and serves as the ground-truth label, providing automatic alignment without manual annotation.

The ABCS dataset contains approximately 42 h of audio from 101 speakers and is officially partitioned into 85 speakers for training, 8 for development, and 8 for testing. The ESMB dataset offers 128 h from 287 speakers. As it lacks an official split, we randomly partitioned the dataset by speaker identity, assigning 240 speakers to the training set, 24 to the development set, and 23 to the test set. This partition was fixed prior to all experiments to maintain consistency, and the detailed speaker split list is publicly available on our project webpage referenced in the Introduction. Crucially, for both corpora, the test sets consist entirely of unseen speakers who do not overlap with the training set. This strictly speaker-independent evaluation protocol ensures that the reported performance reflects the model’s generalization ability to universal acoustic priors rather than overfitting to specific speaker characteristics.

#### 4.1.2. Evaluation Metrics

To provide a comprehensive and systematic assessment of model performance, we employ three sets of standard objective metrics. These metrics were selected to evaluate distinct aspects of restoration quality, including spectral fidelity, intelligibility, and overall perceptual quality, and to ensure fair comparisons with existing state-of-the-art methods.

(1) PESQ (Wide-band) [35]: Based on the ITU-T P.862.2 standard [36], this metric evaluates perceptual speech quality with a range from −0.5 to 4.5. It is particularly appropriate for this task as it assesses the restoration of high-frequency components (up to 7 kHz), which are typically severely attenuated in BC signals.
(2) STOI [37]: This metric measures speech intelligibility by calculating the correlation of short-time temporal envelopes between the clean reference and the enhanced signal (range: 0 to 1). It is essential for verifying that the bandwidth extension process preserves the underlying linguistic content without introducing destructive artifacts.
(3) Composite Metrics [38]: To approximate subjective Mean Opinion Scores (MOSs), we report CSIG (signal distortion), CBAK (background intrusiveness), and COVL (overall quality). These metrics (range: 1 to 5) provide a holistic view of the restoration performance, distinguishing between noise suppression capability and the naturalness of the reconstructed speech signal.

In addition, to evaluate the computational efficiency for potential deployment on edge devices, model complexity is assessed via the number of parameters (Params), model size, and Multiply-Accumulate operations (MACs).

### 4.2. Training Details

#### 4.2.1. Data Preprocessing

Following the standard data preprocessing strategy of Wave-U-Net [34], we utilized the raw waveform directly without applying normalization or overlap-add techniques. During the training phase, input samples were generated by randomly extracting segments of fixed length from the raw recordings, 1 s for the ABCS corpus and 2 s for the ESMB corpus. In cases where the randomly selected segment extended beyond the recording boundary, it was padded with silence to match the target duration. During the inference phase, the full-length audio was processed.

#### 4.2.2. Training Configuration

All models were implemented using Python 3.8 and the PyTorch 1.13.1 framework with CUDA 11.6 and were trained from scratch on a workstation equipped with a single NVIDIA GeForce RTX 4090 GPU (NVIDIA, Santa Clara, CA, USA). We utilized the Adam optimizer with parameters $\beta_{1}=0.9$ and $\beta_{2}=0.999$. The initial learning rate was set to 0.002, with a cosine decay schedule applied after a 50-epoch warm-up period. The models were trained for a maximum of 500 epochs with a batch size of 128. To mitigate the risk of overfitting, we implemented an early stopping mechanism with a 15-epoch patience window, monitoring the sum of PESQ and STOI scores on the development set. The checkpoint yielding the highest composite score was selected for evaluation. Baseline models were trained using their officially recommended configurations to ensure a fair comparison.

#### 4.2.3. Loss Function and Hyperparameters

The proposed model is optimized using the objective function defined in Equation (18). The task-specific loss $L_{task}$ is computed over six window sizes ranging from 64 to 2048 ($2^{6},\ldots ,2^{11}$) with 128 Mel-frequency bins, ensuring robustness across different frequency bands.

For the Key-Value Memory Module, we performed a grid search to determine the optimal hyperparameters. Based on the sensitivity analysis on the development set, we set the number of memory slots N=256 and the temperature scaling factor τ=16. A detailed analysis justifying these selections is provided below.

## 5. Results and Analysis

In this section, we comprehensively evaluate the performance of the proposed method. First, we use the ABCS dataset to conduct an in-depth analysis of the framework’s internal mechanisms, including the impact of key hyperparameters and the contribution of individual modules, to determine the optimal configuration. Subsequently, we compare our proposed method against competitive baselines to validate its superiority in terms of speech quality and intelligibility across both the ABCS and ESMB datasets.

### 5.1. Validation of the Proposed Framework

#### 5.1.1. Impact of Hyperparameters

In our experiments, we first employ the Wav2Vec 2.0 Base model pre-trained on the LibriSpeech [39] corpus as the guidance SSL model. Specifically, rather than using high-level contextualized representations from the Transformer layers, we extract the local latent representations directly from the output of the front-end CNN feature encoder. These low-level embeddings preserve fine-grained acoustic details including pitch and local spectral envelope, which are crucial for reconstructing the waveform structure in time-domain enhancement tasks. Based on this setup, we investigate the sensitivity of two hyperparameters: the number of memory slots *N* and the temperature scaling factor τ as mentioned in Section 3.4.1

**Figure 5:**
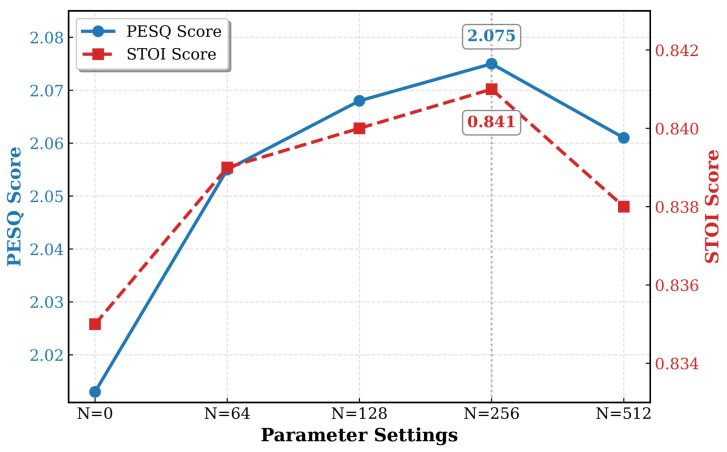
Impact of the number of memory slots *N* on enhancement performance. The performance improves as the memory capacity increases, peaking at N=256, which offers sufficient capacity to cover diverse acoustic prototypes. A decline is observed at N=512, suggesting that an excessively large memory bank may introduce redundancy and noisy features, leading to overfitting.

Figure 5 illustrates the performance trends with varying memory sizes *N*. In this analysis, the parameter τ is fixed at 16, following conventional settings. Specifically, the configuration N=0 denotes the baseline primary Wave-U-Net model, which operates without the guidance of the SSL-derived acoustic priors. It can be observed that the PESQ and STOI metrics exhibit a steady improvement as *N* increases from 0 to 256. This enhancement suggests that a moderately larger memory bank provides sufficient capacity to store a diverse set of acoustic prototypes, thereby covering a wider range of phonemic variations found in clean speech. However, further increasing *N* to 512 leads to a performance degradation. We attribute this decline to redundancy and the risk of overfitting, where an excessively large memory bank may begin to store noisy or indistinguishable features. This redundancy complicates the retrieval process by introducing ambiguity, ultimately reducing the model’s generalization ability. Consequently, N=256 is selected as the optimal configuration.

**Figure 6:**
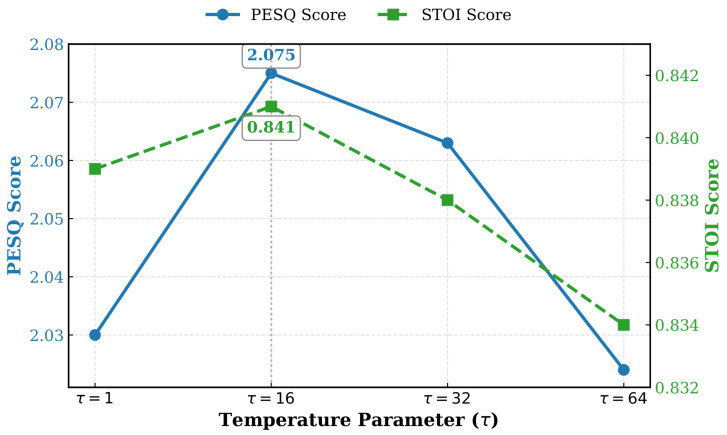
Sensitivity analysis of the temperature parameter τ in the memory addressing mechanism. The results indicate that τ=16 yields the optimal trade-off.

With *N* fixed at 256, we further analyze the impact of the temperature parameter τ, which controls the sharpness of the attention distribution during key-value retrieval. As shown in Figure 6, the performance peaks at τ=16. A value lower than 16 results in an overly flat distribution, causing the retrieved feature to be a blurred average of multiple prototypes, which weakens the semantic guidance. Conversely, an excessively large τ makes the attention distribution too sharp, which may hinder gradient flow and prevent the model from fusing complementary information from multiple relevant slots. Thus, we set τ=16 to achieve the best trade-off between distinctiveness and smoothness.

#### 5.1.2. Effectiveness of Different SSL Configurations

As detailed in Table 2, we evaluated three distinct **Wav2Vec 2.0** models: an English version (trained on LibriSpeech), a Mandarin-Taiwan version (trained on Podcasts [40] and fine-tuned on CommonVoice zh-TW [41]), and a Mandarin-Mainland version (trained on AISHELL-2 [42]). Additionally, we tested a publicly available Chinese **HuBERT** model (Available: https://huggingface.co/TencentGameMate/chinese-hubert-base accessed on 1 February 2026). The feature extraction sources were categorized into “Encoder Feat.” (output of the CNN front-end) and “Context Feat.” (output of the Transformer layers). For the latter, we investigated two extraction strategies: “Context Feat. Last,” which utilizes the representations from the final Transformer layer, and “Context Feat. Avg,” which computes the average of embeddings across all Transformer layers.

The results reveal several key insights:

(1) Impact of Linguistic Consistency: Within the Wav2Vec 2.0 comparisons using Encoder Features, the performance follows the order: Mandarin-Mainland > Mandarin-Taiwan > English. This suggests that while acoustic structures possess some universality, aligning the language of the pre-trained model with the target speech domain yields more precise guidance.
(2) Benefit of Contextual Information: For the Mandarin-Mainland configuration, utilizing *Context Features* yields superior performance compared to *Encoder Features*. This indicates that the high-level semantic and contextual representations learned by the Transformer layers provide robust cues for restoration, surpassing local acoustic features. Regarding HuBERT, extracting embeddings from the *last* Transformer layer yields better results than averaging all layers. We hypothesize that averaging across layers inevitably incorporates corrupted low-level representations, which dilutes the semantic distinctiveness of the retrieval keys. In contrast, the highest semantic layer provides abstract, phoneme-level representations that serve as stable, sensor-invariant anchors, enabling the memory network to accurately look up high-fidelity textures. Consequently, this configuration is adopted as our optimal method.
(3) Scalability with Model Capability: Most notably, the HuBERT-based configuration achieves the best overall performance. HuBERT outperforms its Wav2Vec 2.0 counterpart, demonstrating that our flexible plug-and-play adaptor design allows the restoration framework to scale effectively with stronger upstream SSL models.

#### 5.1.3. Compatibility of the Proposed Framework

To assess the compatibility of our approach, we extended our evaluation to a U-Net-Like backbone, which is designed in [17]. By integrating the optimal HuBERT (Context Feat. Last) embeddings into this alternative structure, we verified that the proposed SSL-based guidance module remains effective across different generator configurations, with detailed results provided in Table 3.

For the proposed framework, removing the SSL module causes a significant performance drop: PESQ declines by 6.68%, with composite metrics ranging from 4.8% to 5.8%, and STOI declines by 2.57%. This confirms that the restoration process relies heavily on pre-trained priors to reconstruct high-frequency details. A similar degradation is observed in the U-Net-Like configuration. These consistent improvements across different backbones validate the SSL module as a versatile plug-and-play component capable of injecting robust acoustic guidance into varying architectures.

#### 5.1.4. Visualization of Memory Mechanism

To gain deeper insights into how the Memory Module encodes and retrieves acoustic information, we visualize the internal statistics of the learned memory slots.

**Figure 7:**
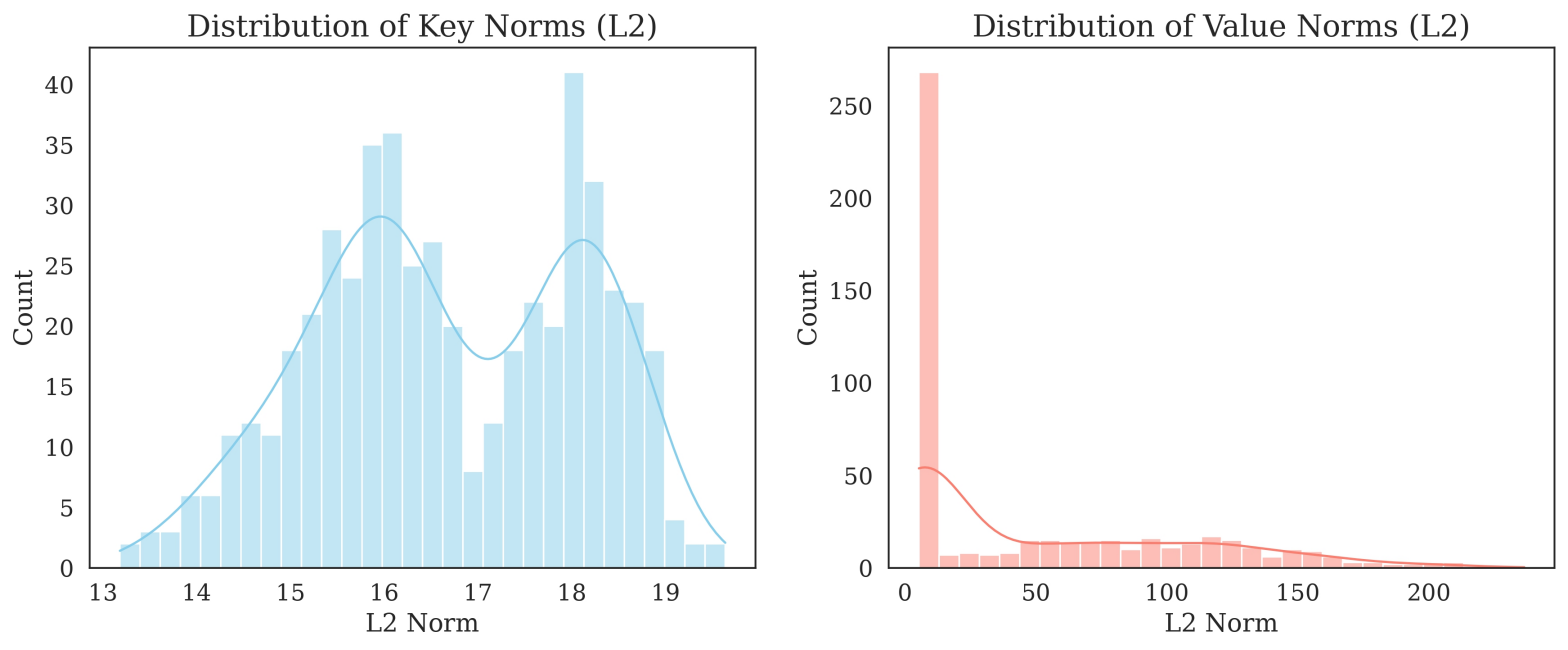
Distribution of L2 norms for Key and Value memory slots. The Key memory follows a normal distribution to facilitate stable query matching. In contrast, the Value memory exhibits a heavy-tailed distribution concentrated near zero, indicating a sparse activation strategy where the model learns to suppress irrelevant slots.

Figure 7 illustrates the distribution of L2 norms for Key and Value memory. While the Key norms follow a relatively normal distribution to facilitate stable querying, the Value norms exhibit a highly skewed, heavy-tailed distribution with a massive concentration near zero. This phenomenon suggests that the model effectively learns a sparse activation strategy. During inference, only a small subset of salient slots contributes significantly to the reconstruction, while the majority of slots remain dormant. This mechanism effectively acts as a noise gate, suppressing ambiguous or irrelevant background signals. Meanwhile, statistical analysis of the Key Memory reveals a 96.88% active utilization rate (addressing weight >1/N) across the test set. Since each key entry is uniquely paired with a value entry, this high utilization confirms that the model retrieves a diverse range of acoustic priors rather than repeatedly accessing a limited subset. This effectively rules out mode collapse and ensures that the rich semantic information stored in the Value memory is fully exploited.

**Figure 8:**
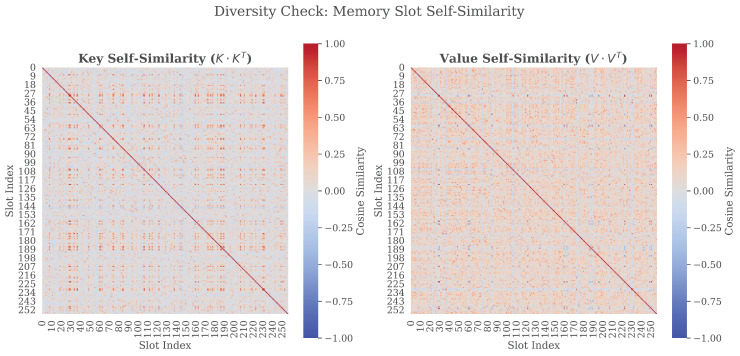
Visualization of self-similarity matrices for memory slots. The Key Memory maintains high orthogonality with a cleaner background for precise addressing, whereas the Value Memory displays a slightly reddish background (similarity ≈ 0.25), reflecting the inherent semantic consistency and acoustic continuity of the stored SSL priors.

We further investigate the redundancy of the memory bank by computing the cosine similarity between all pairs of slots, as shown in Figure 8. In both the Key and Value matrices, we observe a prominent diagonal line set against a background of values approaching zero. The lack of high similarity values in off-diagonal areas indicates that the memory slots are nearly orthogonal to each other. This confirms that the model has successfully learned a diverse set of acoustic prototypes without falling into mode collapse, ensuring that the limited memory capacity covers a wide range of phonemic and spectral variations.

### 5.2. Comparisons with Other Baselines

#### 5.2.1. Baseline Methods

The proposed model is compared with five recent time-domain approaches, including FCN-BC [14], DPT-EGNet [15], EBEN [16], TRAMBA [43], and the U-Net-Like [17]. We utilized the publicly available code for all models, except for U-Net-Like, which was reproduced according to its paper description.

To ensure a fair comparison of model efficiency, we scaled up the first three lightweight models by increasing their network depth and width, creating their larger counterparts denoted as FCN-BC*, DPT-EGNet*, and EBEN*. This was done without altering their core architectural principles.

#### 5.2.2. The Results of Objective Metrics

Table 4 and Table 5 present the quantitative results on the ABCS and ESMB datasets, respectively, where bold values denote the best performance. While most methods show substantial gains over the unprocessed BC speech, FCN-BC exhibits limited improvement due to its simplistic architecture. Interestingly, the relative rankings of DPT-EGNet, TRAMBA, and EBEN diverge across datasets: the latter two excel on ABCS, whereas DPT-EGNet proves superior on ESMB. This is likely attributable to the dual-path Transformer’s enhanced robustness against the complex noise characteristics inherent in the ESMB dataset.

Both the U-Net-Like baseline and our proposed framework demonstrate significant efficacy, affirming the advantage of U-Net-based architectures for this task. Notably, our proposed model achieves state-of-the-art performance, yielding remarkable PESQ improvements of over 51% on the ABCS dataset and 73% on the ESMB dataset compared to the original BC speech.

Table 4 and Table 5 further illustrate the model efficiency. It is observed that simply scaling up the lightweight baselines yields only marginal performance gains, suggesting that their representational capacity is limited by their core architectural design rather than parameter count. In contrast, as shown in Table 6, our proposed model demonstrates a superior efficiency–performance trade-off. With only 3.87 M parameters, it outperforms the much larger U-Net-Like model (9.10 M) while utilizing less than half of its parameters and maintaining lower computational burden (MACs). This confirms that the proposed method achieves high-fidelity enhancement through efficient architectural design rather than brute-force scaling.

#### 5.2.3. Performance Analysis Across Different Genders

**Figure 9:**
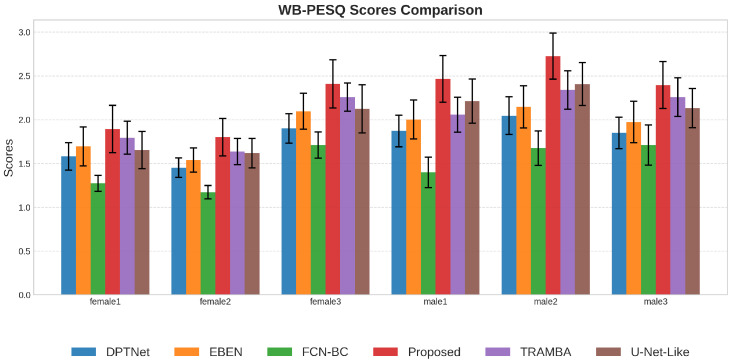
WB-PESQ performance comparison on female and male speakers. The proposed model achieves the best results on all speakers.

**Figure 10:**
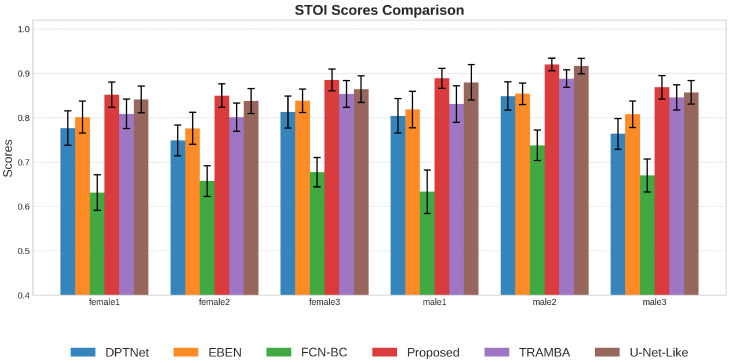
STOI performance comparison on female and male speakers. The proposed method consistently outperforms baselines. This confirms that the incorporated SSL acoustic priors effectively help restore linguistic structures, yielding higher speech intelligibility for both genders.

**Figure 11:**
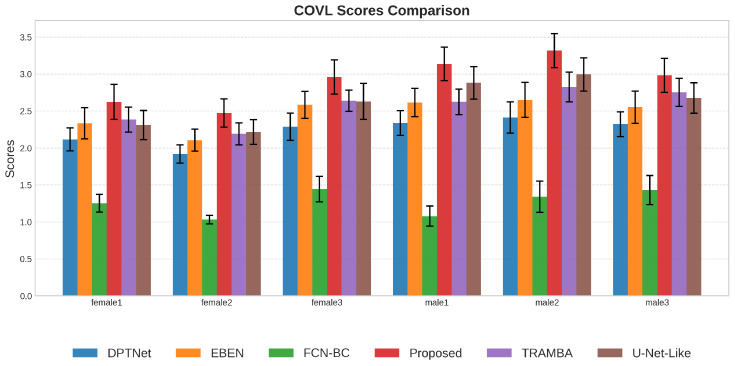
COVL performance comparison on female and male speakers. The proposed framework shows superior generalization capabilities, consistently achieving the highest composite scores compared to other time-domain methods, resulting in more natural-sounding speech.

We further examined the model’s robustness across genders by selecting representative speakers from the test set, as shown in Figure 9, Figure 10 and Figure 11. A general trend observed is that the enhancement quality for female speech is consistently lower than that for males, which can be attributed to the severe loss of high-frequency harmonics in BC signals that disproportionately affects higher-pitched female voices. Despite this inherent physical constraint, the proposed framework demonstrates the most significant resilience, exhibiting the smallest performance degradation among all methods. This advantage is particularly evident in the challenging case of speaker female2, where baseline models like FCN and DPTNet suffer catastrophic failure and become nearly ineffective; in contrast, our model maintains a satisfactory performance level. This confirms that the incorporated SSL priors play a crucial role in compensating for the spectral loss, effectively hallucinating the missing high-frequency details to bridge the quality gap between genders.

#### 5.2.4. Visualization of the Envelopes and Spectrograms

**Figure 12:**
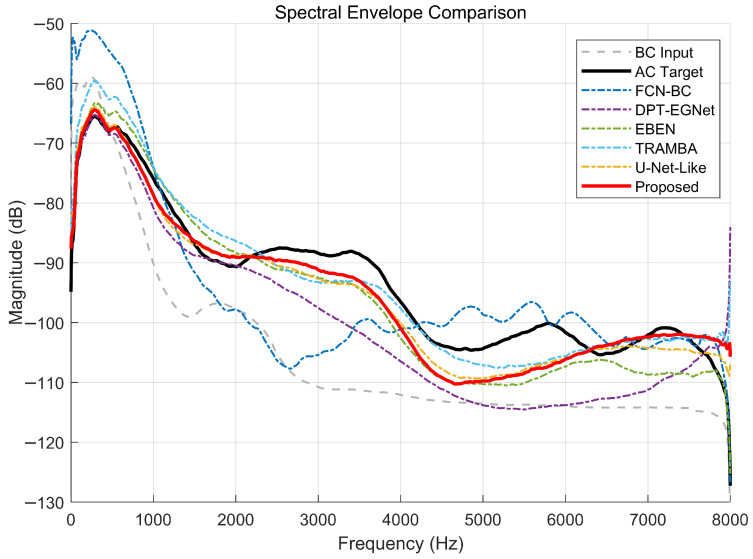
Comparison of spectral envelopes among different enhancement models. The gray dashed curve highlights the severe attenuation of high-frequency components (>1.5 kHz) in the raw BC input. While the FCN-BC baseline exhibits jagged artifacts and spectral distortion, the Proposed method produces a smooth envelope that closely matches the AC Target, effectively recovering the missing high-frequency energy.

**Figure 13:**
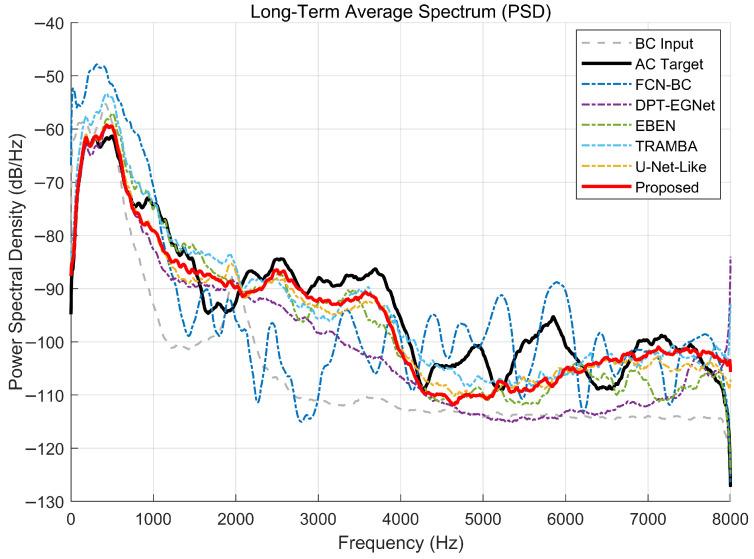
Comparison of Long-Term Average Spectra (PSD) among different models. The proposed model achieves the closest alignment with the AC Target across the entire frequency range. While competitive baselines like EBEN and TRAMBA recover high-frequency energy, the proposed method demonstrates superior stability and envelope fidelity, avoiding the erratic fluctuations observed in FCN-BC and the severe attenuation seen in DPT-EGNet, particularly in the critical 2–4 kHz transition band.

Figure 12 and Figure 13 visualize the Spectral Envelope and Long-Term Average Spectrum (PSD) of the test samples, providing a comprehensive insight into the frequency response characteristics. A critical observation from the gray dashed curves is the severe spectral degradation inherent in BC speech. While the low-frequency energy below 1 kHz is relatively well-preserved, there is a drastic attenuation in the high-frequency band (above 2 kHz), where the signal energy drops nearly to the noise floor. This confirms the strong low-pass filtering effect of the skull and soft tissues, highlighting the extreme difficulty of recovering intelligible speech from such limited high-frequency information.

In terms of reconstruction stability, the baseline models exhibit noticeable limitations. The FCN model, in particular, displays the most unstable performance. As seen in the spectral envelope, the FCN curve suffers from significant distortions, characterized by unnatural jagged oscillations and spurious peaks. This suggests that its simple convolutional architecture struggles to model the global context required for coherent spectral prediction, leading to distinct artifacts and a failure to capture the smooth formant structures of natural speech.

In contrast, the proposed method demonstrates superior fidelity across the frequency range, aligning well with the AC Target. Most notably, in the challenging high-frequency region where the BC input is virtually silent, our model successfully recovers the missing energy and harmonic structures that other baselines often underestimate. Unlike the fluctuating response of FCN-BC, the spectral envelope generated by our method is smooth and consistent with the target, proving that the incorporated SSL priors effectively guide the hallucination of realistic spectral details and ensure a precise recovery of the global energy distribution.

**Figure 14:**
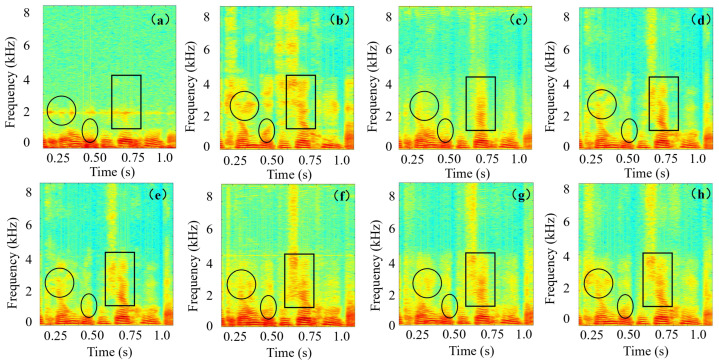
Spectrograms of (**a**) BC speech (**b**) AC speech, and the enhanced results from different models: (**c**) FCN-BC (**d**) DPT-EGNet (**e**) EBEN (**f**) TRAMBA (**g**) U-Net-Like (**h**) Proposed. The regions marked by circles and rectangles highlight critical high-frequency harmonics and unvoiced sounds that are largely lost in the BC input. The proposed method successfully reconstructs these missing spectral components, closely resembling the AC speech reference.

Figure 14 illustrates the spectrograms from different models. As highlighted in the boxes, our proposed method successfully reconstructs fine-grained spectral details that are missing in the original BC speech in (Figure 14a). Crucially, the restored structure bears a much closer resemblance to the ground-truth AC speech spectrogram (Figure 14b), indicating a more accurate and detailed restoration.

#### 5.2.5. Subjective Results

Subjective listening experiments, specifically AB preference tests, were carried out to benchmark the perceptual fidelity of our proposed framework against established baseline methods. Five pairs of listening tests were conducted: Proposed versus FCN-BC, DPT-EGNet, EBEN, TRAMBA, and U-Net-Like. For each test, 20 listeners were presented with 16 paired audio samples in random order and required to choose the speech with better quality or select the “Fair” option if no significant difference was perceived. To quantify the statistical reliability, a two-sided binomial test was performed by comparing the number of votes for the “Proposed” method against the “Baseline” method to verify if the preference was significant.

**Figure 15:**
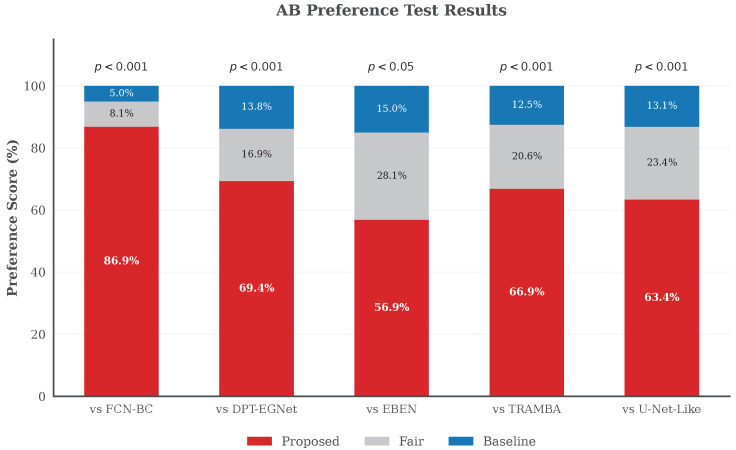
AB preference test results among different methods. The proposed model (red bars) consistently achieves the highest preference rates. Statistical analysis confirms this advantage, with all comparisons yielding *p*-values below 0.05, and the vast majority reaching a high significance level of p<0.001.

Figure 15 presents the results of the AB preference test, annotated with statistical significance levels (*p*-values). Our proposed model achieves the highest preference rate across all comparison pairs with high statistical significance, demonstrating its superior capability in generating natural and high-fidelity speech. Specifically, the proposed method overwhelmingly outperforms the FCN-BC baseline with an 86.9% preference rate and maintains a significant lead over DPT-EGNet and TRAMBA.

Notably, the comparison with EBEN reveals an interesting phenomenon. While EBEN typically yields lower objective scores in previous quantitative experiments, it exhibits the most competitive performance here, with the highest “Fair” rate of 28.1% and a comparatively lower win rate for our model. This discrepancy can be attributed to EBEN’s GAN-based architecture, which prioritizes the generation of perceptually plausible signal distributions over the minimization of point-wise reconstruction errors. Consequently, EBEN produces speech with high perceptual quality that appeals to human listeners, even if it is not favored by standard objective metrics. Nevertheless, our model still outperforms EBEN, confirming the effectiveness of the proposed memory-augmented SSL guidance strategy.

## 6. Conclusions

This paper presents a novel time-domain framework to address the fundamental bandwidth limitations of BC signals. Our approach successfully leverages generalized acoustic representations from self-supervised models and integrates them as restoration cues via a key-value memory network to compensate for physical information scarcity. A critical innovation lies in the ability of the memory network to bridge the sensor domain gap, enabling the retrieval of idealized acoustic priors during inference without requiring reference AC signals. Furthermore, the proposed architecture follows a plug-and-play design, allowing for the flexible integration and upgrading of various backbones and pre-trained models depending on the specific sensor requirements.

Extensive experiments validated the efficacy of this paradigm in restoring fine-grained spectral details physically attenuated by the human body. The proposed model establishes a new state-of-the-art by decisively outperforming recent time-domain approaches in both subjective and objective metrics. Crucially, despite the computational overhead associated with the SSL-based training loop, the deployed model is structurally decoupled from these heavy backbones. Consequently, it retains a lightweight footprint of only 3.87 M parameters, facilitating practical implementation on resource-constrained edge platforms. Our detailed analysis further revealed that the quality of restoration cues depends on the linguistic proximity and the specific extraction layer of the pre-trained model. Additionally, the learned memory slots demonstrate high orthogonality, which ensures efficient and distinct representation retrieval. Driven by this balance of high performance and efficiency, the proposed framework holds vast potential for real-world deployment, particularly in wearable devices, assistive hearing technologies, and critical communication systems operating in noisy environments.

In future work, we aim to address remaining challenges regarding model generalization and precision. We will focus on optimizing the key-value memory network to more accurately mimic idealized priors and explore lightweight, task-specific adaptation of the frozen pre-trained models to ensure optimal alignment with BC sensor requirements. Additionally, we plan to extend our evaluation to more diverse scenarios, specifically investigating the model’s robustness under varying sensor wearing positions and lower signal-to-noise ratio conditions, to further validate its generalization ability in complex real-world environments.

## Figures and Tables

**Table 1:** Detailed architecture of the Mainstream Module. *n* represents the base number of convolutional channels, and *i* denotes the layer index.

Block	Operation	Input Shape (1 s)	Input Shape (2 s)
Input	-	(16,384, 1)	(32,768, 1)
Encoder (i=1,…,8)	Conv1D (n×i) Decimation	(64, 200)	(128, 200)
Fusion Layer	8-head Cross-Attention	(64, 200)	(128, 200)
Decoder (i=8,…,1)	Upsample Concat (Skip Conn.) Conv1D (n×i)	(16,384, 25)	(32,768, 25)
Output	Concat (Raw Input) Conv1D (1)	(16,384, 26) (16,384, 1)	(32,768, 26) (32,768, 1)

**Table 2:** Performance comparison of different SSL model configurations on the test set.

Model & Configuration	PESQ	STOI	CSIG	CBAK	COVL
**Wav2Vec 2.0**					
English (Encoder Feat.)	2.075	0.841	3.369	2.477	2.681
Mandarin-Taiwan (Encoder Feat.)	2.089	0.843	3.376	2.498	2.707
Mandarin-Mainland (Encoder Feat.)	2.096	0.845	3.387	2.502	2.715
Mandarin-Mainland (Context Feat. Last)	2.128	0.855	3.439	2.531	2.763
**HuBERT**					
Mandarin-Mainland (Encoder Feat.)	2.146	0.855	3.433	2.533	2.762
Mandarin-Mainland (Context Feat. Avg)	2.154	0.856	3.449	2.539	2.773
Mandarin-Mainland (Context Feat. Last)	**2.157**	**0.857**	**3.452**	**2.545**	**2.784**

**Table 3:** Effectiveness of the SSL-based module and its plug-and-play capability across different architectures.

Configuration	PESQ	STOI	CSIG	CBAK	COVL
**Proposed**	2.157	0.857	3.452	2.545	2.784
- w/o SSL model	2.013	0.835	3.285	2.421	2.624
(Percentage Drop)	(−6.68%)	(−2.57%)	(−4.84%)	(−4.87%)	(−5.75%)
**Proposed (U-Net-Like)**	2.024	0.846	3.259	2.047	2.573
- w/o SSL model	1.924	0.827	3.124	1.964	2.452
(Percentage Drop)	(−4.94%)	(−2.25%)	(−4.14%)	(−4.05%)	(−4.70%)

**Table 4:** Comparisons with recent Time-Domain models on the ABCS dataset.

Model	PESQ	STOI	CSIG	CBAK	COVL
BC Speech	1.425	0.691	2.087	1.560	1.677
FCN-BC	1.677	0.660	2.323	2.081	2.085
FCN-BC*	1.697	0.671	2.353	2.112	2.121
DPT-EGNet	1.799	0.789	3.001	2.357	2.376
DPT-EGNet*	1.953	0.831	3.083	2.461	2.499
EBEN	1.833	0.793	3.221	2.475	2.485
EBEN*	1.874	0.796	3.336	2.492	2.516
TRAMBA	1.985	0.819	3.095	2.169	2.511
U-Net-Like	1.924	0.827	3.124	1.964	2.452
**Proposed**	**2.157**	**0.857**	**3.452**	**2.545**	**2.784**

**Table 5:** Comparisons with recent Time-Domain models on the ESMB dataset.

Model	PESQ	STOI	CSIG	CBAK	COVL
BC Speech	1.024	0.420	1.017	1.004	1.003
FCN-BC	1.095	0.441	1.023	1.016	1.013
FCN-BC*	1.105	0.445	1.033	1.021	1.033
DPT-EGNet	1.635	0.645	3.171	2.039	2.413
DPT-EGNet*	1.658	0.674	3.260	2.051	2.522
EBEN	1.309	0.612	2.761	1.856	2.003
EBEN*	1.383	0.628	2.799	1.863	2.039
TRAMBA	1.329	0.569	2.965	1.747	2.104
U-Net-Like	1.618	0.683	3.212	2.019	2.399
**Proposed**	**1.771**	**0.695**	**3.366**	**2.233**	**2.573**

**Table 6:** Model complexity comparison.

Model	Params (M)	Model Size (MB)	MACs (G)	
			ABCS (1 s)	ESMB (2 s)
FCN-BC	0.01	0.04	0.14	0.28
FCN-BC*	3.91	15.64	3.89	7.78
DPT-EGNet	0.52	2.08	3.85	7.70
DPT-EGNet*	3.96	15.84	26.25	52.50
EBEN	1.98/29.70 ^a^	7.92/118.80 ^a^	1.02	2.04
EBEN*	5.38/33.10 ^a^	21.52/132.40 ^a^	3.10	6.20
TRAMBA	5.20	20.80	0.57	1.14
U-Net-Like	9.10	36.40	3.32	6.64
**Proposed**	3.87	15.48	2.43	4.74

^a^ EBEN is a GAN-based architecture; parameters are for the generator (inference) and the full model (total learnable).

## Data Availability

Publicly available datasets were analyzed in this study. The Air- and Bone-conduction Synchronized (ABCS) corpus can be found here: https://github.com/wangmou21/abcs (accessed on 1 February 2026). The Elevoc Simultaneously recorded Microphone/Bone-sensor (ESMB) corpus can be found here: https://github.com/elevoctech/ESMB-corpus (accessed on 1 February 2026).
